# Case Report: A role for hemoadsorption in hemophagocytic lymphohistiocytosis

**DOI:** 10.3389/fmed.2025.1729061

**Published:** 2025-12-18

**Authors:** Miguel Gonçalves Pereira, César Burgi Vieira, Tiago Isidoro Duarte, Nuno Germano

**Affiliations:** 1 Department of Intensive Care Medicine, Unidade Local de Saúde da Guarda, Hospital Sousa Martins, Guarda, Portugal; 2 Department of Intensive Care Medicine, Unidade Local de Saúde de São José, Hospital Curry Cabral, Lisbon, Portugal

**Keywords:** hemophagocytic lymphohistiocytosis, secondary HLH, cytokine storm, hemoadsorption, CytoSorb^®^, extracorporeal blood purification, continuous renal replacement therapy, critical care

## Abstract

Hemophagocytic lymphohistiocytosis (HLH) is a rare but life-threatening hyperinflammatory syndrome. HLH can occur as a primary (familial) or secondary (acquired) disorder. Secondary HLH (sHLH) manifests in response to infection, malignancy, autoimmune disorders or drugs. Presentation is usually non-specific, with fever, cytopenias, elevated inflammatory markers and hepatosplenomegaly. A high level of suspicion coupled with the use of diagnostic scores (HScore and HLH-2004) is required for timely diagnosis. Specific studies such as bone marrow biopsy or soluble interleukin-2 (IL-2) receptor (CD25) levels are necessary for distinguishing HLH from other conditions, but should not delay treatment. Corticosteroids, in addition to other immunossupressants, should be initiated promptly in order to reduce morbidity and mortality. In secondary cases, treatment of underlying causes is necessary for complete remission. Long-term immunossupression may be required beyond resolution of the HLH trigger to avoid relapses. We report a case of severe acute HLH in a patient with distributive and cardiogenic shock and multiorgan dysfunction. The patient was successfully treated with CytoSorb^®^ hemoadsorption therapy. This case highlights diagnostic challenges, therapeutic interventions, and the potential role of hemoadsorption therapy as a complementary treatment option for HLH.

## 1 Introduction

Hemoadsorption is an extracorporeal technique that attempts to reduce hyperinflammatory states by removing circulating cytokines and other hydrophobic molecules. The CytoSorb^®^ device uses biocompatible porous polymer beads that adsorb molecules between 5 and 60 kDa, which includes key mediators in HLH. By lowering the circulating cytokine burden, without adding pharmacological immunosuppression, hemoadsorption might work as a useful adjunct in infection-triggered HLH - a context where conventional immunosuppression can make the underlying infection worse. Its use in HLH is still quite new. Evidence comes mostly from case reports, but there’s emerging data suggesting potential benefit when cytokine storm syndromes evolve rapidly. This case adds clinical information to this developing therapeutic approach.

Hemophagocytic lymphohistiocytosis (HLH) is a rare but life-threatening hyperinflammatory syndrome. HLH is characterized by a cytokine burst, such as interleukin 6 (IL-6), interferon-gamma (IFN-gamma) and CD25 (1), associated with macrophage and NK- cell activation (2). HLH can occur as a primary (familial) or secondary (acquired) disorder. Secondary hemophagocytosis is the most frequent form in adults and is often in response to an infection, usually involving immune dysfunction, autoimmune conditions, malignancies or even medications (3). HLH manifests clinically with nonspecific symptoms such as fever, hepatosplenomegaly, cytopenias and elevated inflammatory markers. Despite the advances in understanding HLH, delays in the diagnosis and treatment remain common. Not only is it a rare condition, HLH is also difficult to distinguish from other severe systemic inflammatory conditions (4). Prompt recognition and initiation of immunosuppressive therapy are critical to improving patient outcomes (5).

Over the years, several scoring systems (Table 1) have been developed to help with both the diagnosis and prognosis. Among them, the HScore and HLH-2004 criteria are the most commonly used. The HScore assigns a probability of HLH based on a combination of clinical, laboratory, and histological findings, making it particularly useful in adult patients. In contrast, the HLH-2004 criteria, originally designed for primary HLH, require at least five out of eight specific features, such as persistent fever, cytopenias, hyperferritinemia, and hemophagocytosis in bone marrow. While these tools are valuable, both have limitations and may not fully account for the syndrome’s variability, emphasizing the importance of a comprehensive clinical assessment to ensure timely and accurate diagnosis.

**Table 1:** Hemophagocytic lymphohistiocytosis scores and criterias.

Criteria	Hscore	HLH-2024	2016 sJIA MAS	MS score
Fever (°C)	0 (<38.4 °C), 33 (38.4–39.4 °C) or 49 (>39.4°C)	≥38.5	Degree not specified	NA
Ferritin	0 (<2,000), 35 (2,000–6,000) or 60 (>6,000)	≥500	>684	0.0001 × serum level
Hematologic	0 (1 lineage), 24 (2 lineages) or 34 (3 lineages)^1^	Two or three out of three lineages^2^	Platelets ≤181 × 109 /L	−0.003 × platelet count
Organomegaly	0 (no), 23 (hepatomegaly or splenomegaly) or 38 (hepatomegaly and splenomegaly)	Splenomegaly	NA	NA
Triglyceride	0 (<1.5 mmol/L), 44 (1.5 – 4 mmol/L) or 64 (>4 mmol/L)	≥265 mg/dL^3^	>156 mg/dL	NA
Hemorrhagic	NA	NA	NA	1.54 (yes) or 0 (no)
Fibrinogen (g/L)	0 (>2.5) or 30 (<2.5)	≤1.5	≤360 mg/dL	−0.004 × serum level
LDH	NA	NA	NA	0.001 × serum level
AST (IU/L)	0 (<30) or 19 (>30)	NA	>48 units/mL	NA
CNS involved	NA	NA	NA	2.44 (yes) or 0 (no)
Arthiritis active	NA	NA	NA	−1.3 (yes) or 0 (no)
sCD25 (units/mL)	NA	≥2,400	NA	NA
Known immune- supression	0 (no), 18 (yes)	NA	NA	NA
Pathology	Hemophagocytosis: 0 (no), 35 (yes)	Hemophagocytosis	NA	NA
NK cell activity	NA	Low or absent	NA	NA
Diagnosis	Sum of score >169	Five of eight criteria	Fever + sJIA + elevated ferritin + two of four criteria	Sum ≥ − 2.1

^1^Hemoglobin < 9.2 g/dL, platelets <110 × 10^9^/L, leukocytes <5.0 × 10^9^/L. ^2^Hemoglobin < 9.0 g/dL, platelets <100 × 109/L, neutrophils <1.0 × 109/L. ^3^Either high triglyceride and/or low fibrinogen (counts as one criterion). AST, aspartate aminotransferase; CNS, central nervous system; IU, international units; LDH, lactate dehydrogenase; MAS, macrophage activation syndrome; MS, MAS/systemic juvenile idiopathic arthritis; NA, not applicable; NK, natural killer; sCD25, soluble interleukin-2 receptor α chain; sJIA, systemic juvenile idiopathic arthritis.

Herein, we report a case of severe acute HLH in severely immunosuppressed patient from HIV/TB co-infection, presenting with distributive and cardiogenic shock with multiorgan dysfunction, successfully treated with cytokine hemoadsorption therapy. We highlight the main clinical presentations, diagnostic challenges and therapeutic interventions. By presenting this case, our main goal is to contribute to the growing body of literature on HLH, emphasizing the importance of clinical awareness in recognizing this entity with high morbimortality, as well as the potential application of hemadsorption therapy in the treatment of hyperinflammatory syndromes such as HLH.

## 2 Case report

A 56-year-old female patient, originally from Guinea-Bissau, presented to the emergency department with a two-week history of fever, asthenia, adynamia, extremity tremors, and psychomotor impairment. Relevant past medical history included type 2 diabetes mellitus, dyslipidemia, and adenomatous thyroid hyperplasia. The initial work-up was inconclusive and the patient was admitted to the infectious diseases (ID) unit for further etiological investigation. A previously unknown CDC stage C3 human immunodeficiency virus (HIV) infection was diagnosed, with a viral load of 7 × 10^6^ copies/ml and a CD4 count of 124 cells/μL. Full-body contrast-enhanced CT findings were suggestive of military tuberculosis with pulmonary, intestinal, and renal involvement- supported by positive nucleic acid amplification testing (NAAT) and acid-fast bacilli (AFB) detection in bronchoalveolar lavage fluid. Subsequent cranial contrast-enhanced computed tomography (CT) and magnetic resonance imaging (MRI) were unremarkable. Cerebro-spinal fluid (CSF) cytochemical and microbiological exams were negative.

Due to rapid clinical deterioration in the first 48 h after admission, with severe hypotension, tachycardia, tachypnea, and altered mental status, the patient was admitted to the intensive care unit (ICU) due to respiratory, circulatory and renal failure, requiring mechanical ventilation (MV), vasopressor support and continuous renal replacement therapy. After consultation with ID, empirical antibiotherapy with vancomycin, meropenem and caspofungin was initiated, as well as treatment for miliary tuberculosis with isoniazid, rifampin, ethambutol and levofloxacin. Ganciclovir was also administered due to cytomegalovirus viremia (736 copies/mL). Blood cultures were positive for strains of *Staphylococcus epidermidis* and *Pseudomonas aeruginosa* susceptible to the empirical antibiotic regimen. Despite adequate treatment, the patient’s condition deteriorated, with persistent fever and refractory circulatory and cardiogenic shock. Laboratory results showed worsening cytopenias and elevated ferritin and IL-6 levels. Based on the clinical and analytical findings, a suspicion of secondary hemophagocytic syndrome associated with HIV and disseminated tuberculosis was raised, with an HScore of 188 points (70–80% probability) (Tables 1, 2).

**Table 2:** H-score criteria.

The diagnosis of HLH can be established if criterion 1 or 2 is fulfilled
1. A molecular diagnosis consistent with HLH
2. Diagnostic criteria for HLH fulfilled (5 of the 8 criteria below): - Fever - Splenomegaly - Cytopenias - Hypertrigliceridemia - Hypofibrinogemia - Low or no NK cells activity - Ferritin >500 ug/L - sCD25 (i.e., soluble IL2 receptor) > 2,400 U/mL - Hemophagocytosis in bone marrow or spleen or lymph nodes

To address the hemophagocytic syndrome, corticosteroid therapy with methylprednisolone at 1 g/kg/day was administered. In addition, combined continuous renal replacement therapy and hemoadsorption therapy was performed in continuous veno-venous hemodiafiltration mode using a heparin-based anticoagulation protocol supplemented by a CytoSorb^®^ adsorber (CytoSorbents Corporation, New Jersey, United States), installed in-line in the CRRT circuit (multiFiltratePRO with Ultraflux AV1000S polysulfone membrane, Fresenius Medical Care, Bad Homburg, Germany) in a pre-dialyzer position. Blood flow rates were kept between 200 and 250 mL/min while dialysis doses ranged between 25 and 30 mL/kg per hour, according to routine procedure using the CytoSorb^®^ cartridge (6). A total of approximately 56 h of hemoadsorption with CVVHDF was performed.

Following the initiation of hemoadsorption therapy, the clinical condition recovered remarkably, allowing for vasopressor tapering and MV weaning. Furthermore, inflammatory biomarker levels showed significant improvement (Table 3).

**Table 3:** Laboratory evolution.

Variable/Days in ICU	Day 0	Day 1	Day 2	Day 3	Day 4	Day 5	Day 6	Day 7	Day 8	Day 9	Day 10
Leucocytes (×10^9^/L)	14.1	17.4	11.2	9	6.2	7.8	6.5	5.3	4.9	5.7	8.5
Lactate (mmol/L)	4.0	9.6	<1.5	<1.5	<1.5	<1.5	<1.5	<1.5	<1.5	<1.5	<1.5
Interleukin-6 (pg/mL)	>50,000	285	53.3	–	–	–	–	–	–	–	–
Triglycerides (mg/dL)	452	594	–	492	482	293	288	314	375	–	357
Ferritin (ng/mL)	38,303	47,565	–	20,003	10,341	6,155	5,687	5,957	5,522	6,030	5,785
CD25 (pg/mL)	14,250	–	–	–	–	–	–	–	–	–	–
Noradrenaline (mcg/kg/min)	0.6	1.2	0.13	0.04	0	0	0	0	0	0	0
Methylene Blue (mg/kg/h)	0.3	0.3	0	0	0	0	0	0	0	0	0
Dobutamine (mcg/kg/min)	0	5	0	0	0	0	0	0	0	0	–
SOFA	4	–	–	–	13	11	7	9	7	–	–
CRRT	Start	Yes	Yes	Yes	Yes	No	No	No	No	No	No
CYTOSORB^®^	Start	Yes	Yes	No	No	No	No	No	No	No	No

From day 0 (admission in ICU) to day 2, correspond to period of CytoSorb^®^ adsorbent application through CVVHDF. A dash (–) indicates absence of data for that variable on the respective day, either due to non-performance or unavailability of the measurement.

## 3 Discussion

HLH is a rare and life-threatening hyperinflammatory syndrome, that is characterized by a cytokine burst and usually involves a dysregulated immune response. Secondary HLH is the most frequent form in adults and often occurs in response to an infection.

A final diagnosis of HLH hinges on high clinical suspicion and pattern recognition. Due to the exceedingly high mortality related to HLH, there is a high risk of missing the diagnosis. Therefore, it needs to be balanced against potential side-effects from aggressive immunosuppressive therapy. Early treatment should be considered if there is a reasonable clinical suspicion. The decision to start treatment depends largely on expert opinion and clinical experience, as evidence-based guidelines are not available. Based on available evidence and according to systematic reviews, we suggest a treatment approach for sHLH (Table 4). Note that the recommended therapy with steroids and etoposide is not appropriate in all cases and is associated with considerable risks.

**Table 4:** Treatment approach for secondary HLH.

Timing	Choice of agent	Dose	Considerations
First-line (consider combination therapy)	Anakinra	1–2 mg/kg/dose SC (max 100 mg) q12h; can be increase to max 8 mg/kg/day	Preferred over steroids if the trigger is not clear; consider high starting dose and tapering regimen.
	Methylprednisolone	30 mg/kg/dose IV (max 1 g) q24h, for 3 days	May mask lymphoma diagnosis; Consider Dexamethasone in CNS involvement
	Intravenous immunoglobulin	2 g/kg/dose over 1–2 days	Consider repeat dose after 14 days
Second-line	Etoposide	- 100 mg/m^2^ once weekly in older teens; - 75 mg/m^2^ once weekly in adults - 50 mg/m^2^ once weekly in elderly	Specially in malignancy-related sHLH. Dose reduction in renal and liver failure, hypoalbuminemia, hyperbilirubinemia
Maintenance/relapse prevention	Ciclosporin	1–2 mg/kg/day enterally or IV, then titrated to target levels	May be considered as second-line treatment in some patients
Parallel treatment considerations (examples)	Rituximab		EBV related lymphoproliferative disease
	HAART		HLH secondary to HIV
	Antituberculosis		HLH secondary to Tuberculosis
	Hemoadsorption		Early initiation and patient selection appear to be key factors influencing efficacy

In this case, the presence of fever, bicytopenia, hyperferritinemia, and hypercholesterolemia raised the suspicion of hemophagocytic syndrome, leading to a full diagnostic workup. As an underlying infectious cause was present (HIV infection and a miliary tuberculosis), the patient was considered to have acquired HLH. The combination of septic shock and immunosuppression led to an overwhelming inflammatory response and a potentially fatal cytokine storm (7). Immunosuppression with corticosteroids and anakinra would be warranted. However, the risk of additional immunosuppression was considered too high after consultation with ID and Hematology. For that reason, hemadsorption with the CytoSorb® adsorber was considered a valid complementary treatment as a salvage therapy since it may remove both inflammatory cytokines and HLH cytokines (8).

Several important studies have highlighted the potential of hemoadsorption as an effective therapy in septic shock, but also in critically ill patients without septic shock (9–11). When compared to standard continuous veno-venous hemodiafiltration (CVVHDF), hemoadsorption has been shown to efficiently remove inflammatory cytokines, lowering the need for high-dose vasopressor infusions, and rapidly decrease procalcitonin (PCT) and C-reactive protein (CRP) levels (12, 13). In spite of these promising results, the effectiveness of CytoSorb^®^ therapy in septic shock has yet to be established through randomized controlled trials (RCTs). Although the two available RCTs showed significant reductions in inflammatory cytokines within the treatment groups, these findings did not translate into improved clinical outcomes (14, 15). These findings may be related to the heterogeneity of clinical phenotypes in septic shock trials.

Given the rarity of HLH, available data are limited. Cytokines are known to play a key role in the pathogenesis of HLH, contributing to higher mortality. This provides a strong pathophysiological basis for early application of hemoadsorption in these patients. However, evidence on the impact of cytokine removal on morbidity and mortality is lacking, particularly in such a rare condition such as HLH. Current understanding is primarily derived from isolated case studies (16, 17).

HLH associated with sepsis presents a unique treatment challenge, as the strong immunomodulation required for HLH management may exacerbate sepsis. This case report suggest hemadsorption may have a role in hyperinflammatory states such as HLH, particularly when additional immunosuppression may be harmful. However, further research is needed to validate these findings and to better understand the potential impact of CytoSorb^®^ hemoadsorption therapy on HLH.

Hemoadsorption works by integrating an adsorptive cartridge into an extracorporeal circuit, usually CRRT. The CytoSorb^®^ cartridge contains polystyrene–divinylbenzene beads with controlled pore sizes that allow hydrophobic adsorption of molecules (mostly in the 5–60 kDa range). This includes most cytokines involved in HLH pathogenesis (IL-6, IL-18, IFN-*γ*, TNF-*α*, sCD25). While larger molecules get only partially adsorbed, smaller ones are barely affected.

In our patient the device was placed pre-filter in a CVVHDF circuit with standard blood flows (200–250 mL/min), which allowed simultaneous renal support and kept the adsorptive performance stable. The rapid drop in IL-6 (from >50,000 pg./mL down to 285 pg./mL in 24 h), ferritin reduction and early hemodynamic stabilization matches what’s been seen in septic shock and COVID-19 studies (10, 11, 13). Our patient had profound immune dysfunction (HIV/TB) and some of the highest cytokine levels we have seen reported, but still showed marked improvement after hemoadsorption. Importantly, the technique allowed us to control hyperinflammation without adding more pharmacological immunosuppression—which would’ve been dangerous given the disseminated TB.

This case adds useful data to the limited literature on hemoadsorption in HLH and shows it might work as rescue therapy in severe infection-triggered cytokine storm, where intensified immunosuppression is undesirable.

## Data Availability

The original contributions presented in the study are included in the article/supplementary material, further inquiries can be directed to the corresponding author.
